# Mantle upwelling beneath the South China Sea and links to surrounding subduction systems

**DOI:** 10.1093/nsr/nwz123

**Published:** 2019-08-28

**Authors:** Jian Lin, Yigang Xu, Zhen Sun, Zhiyuan Zhou

**Affiliations:** 1 Department of Geology and Geophysics, Woods Hole Oceanographic Institution, USA; 2 Key Laboratory of Ocean and Marginal Sea Geology, South China Sea Institute of Oceanology, Chinese Academy of Sciences, China; 3 State Key Laboratory of Isotope Geochemistry, Guangzhou Institute of Geochemistry, Chinese Academy of Sciences, China

The evolution of the South China Sea (SCS) is directly linked to the complex subduction systems of the surrounding Pacific, Philippine Sea and Indo-Australian Plates (Fig. 1a). Major advances in the last several years are providing new insights into the SCS-mantle dynamics, through regional seismic imaging of the upper mantle [1,2], unprecedented IODP drilling expeditions (349/367/368/368X) [3–5] that obtained the oceanic basement basalt samples for the first time, geochemical analyses of the SCS-mantle source compositions [6–8] and geodynamic modeling [9,10]. Furthermore, new geological mapping, seismic imaging [11,12] and IODP drilling [13,14] have revealed evidence for significantly greater magma production at the northern SCS rifted margin, in comparison to the magma-poor end-member of the Atlantic rifted margins. This paper provides a new perspective of the SCS-mantle dynamics inspired by new observations and geodynamic modeling. We first highlight new geophysical evidence for a broad region of low-seismic-velocity anomalies in the upper mantle beneath the northern SCS, abundant magmatism during continental breakup and post-seafloor spreading, and geochemical evidence for recycled oceanic components beneath the SCS. We then present new models of layered flows in the mantle beneath the SCS, revealing two modes of plate- and subduction-driven mantle upwelling, including (i) narrow centers of mantle upwelling at shallow depths induced by divergent plate motion at seafloor-spreading centers and (ii) broad zones of mantle upwelling as a result of subduction-induced mantle-return flows at greater depths. These new observations and geodynamic studies suggest strong links between mantle upwelling beneath the SCS and surrounding subducting plates.

## Broad zone of seismic anomalies in the upper mantle beneath the northern SCS

Several new regional seismic studies, using both teleseismic travel-time inversion and receiver function analysis, have revealed strong evidence for seismic velocity anomalies in the upper mantle beneath the northern SCS. The mantle transition zone (MTZ) beneath Hainan Island and the Leizhou Peninsula is observed to be 40–50 km thinner than the global average, suggesting higher-than-average temperatures of ~270–380°C and ~200–240°C, respectively, at the 660- and 410-km discontinuities [15,16]. However, the region of the observed MTZ thinning is relatively narrow, at only ~400 km in width [15].

In contrast, the region of low P-wave anomalies above the 410- to 660-km MTZ is much broader, extending northeastward from the Hainan region over 1600 km (Fig. 2a) [2]. Below the MTZ, however, the P-wave anomalies become much weaker and shifted significantly to the southeast from Hainan Island (Fig. 2a). Thus, the observed broad P-wave anomalies above the MTZ, together with diminishing and shifting anomalies below the MTZ, challenge the view of a ‘Hainan plume’ as a classical fixed narrow thermal plume that originated from the core–mantle boundary. However, for the vast region beneath the SCS ocean basins, direct constraints on the upper-mantle seismic structure are still lacking, which should be an important area of future investigation.

**Figure 1:**
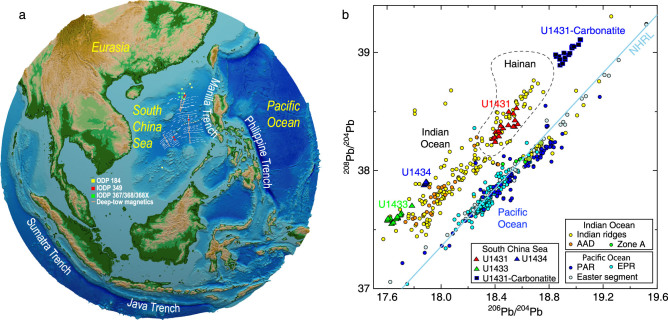
(a) Tectonic map showing that the SCS is surrounded by major subduction systems. Locations of ODP/IODP drill sites and deep-tow magnetic survey lines are shown. (b) Isotope geochemistry of the SCS from IODP Sites U1431, U1433 and U1434 showing the dominance of Indian Ocean-type mantle source. The new SCS data are from Refs [6,7], while the Indian/Pacific data are from Ref. [40].

**Figure 2:**
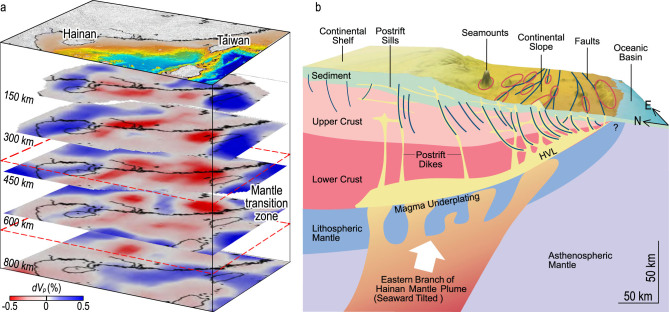
(a) Broad zone of mantle seismic anomalies down to a depth of 800 km beneath the SCS based on data of [2]. (b) A 3D schematic diagram showing a possible mechanism of voluminous post-spreading seamount formation modified from Ref. [11] (permission of its use obtained from John Wiley and Sons with license number of 4658621236510).

## Large volume of post-spreading magmatism

Recent geophysical studies suggest that the SCS and Indochina Peninsula might contain large volumes of post-spreading magmatism in the form of seamounts, eruption, intrusion and underplating [11,12,17–19]. The integrated volume of the 109 seamounts in the whole SCS was calculated to be ~9551 km^3^, while the total volume of the intrusive magma above Moho was estimated to be ~0.15 Mkm^3^ [11], which is surprisingly large and is similar to the median value of the global large igneous provinces (LIPs) [20]. Another study yields an even greater seamount volume [19]. Together with the above evidence for a broad region of low-seismic-velocity anomalies above the MTZ, it is hypothesized that the northern SCS might be considered as a unique province of broad mantle upwelling (Fig. 2b) [11,12], i.e. the South China Sea Mantle Upwelling Province (SCS_MUP).

The timing of the SCS magmatism is, however, still poorly known. Limited seafloor drilling and rock dredging indicate an age span of 17–23 Ma for seamounts in the northeastern continental margin [11]. Seamounts in the oceanic basins are much younger with an age span of 3–15 Ma [21]. Magmatism in the southern Indochina Peninsula shows multi-stage eruptions from ~17.6 Ma to recently [22]. Magmatism around Hainan Island also shows multi-stage eruptions with an age span of 0.6–13 Ma and a peak age of <4 Ma [23]. Overall, the lack of robust age constraints on magmatism still leaves significant uncertainties in the rates of post-spreading magmatism and thus should be another important direction for future research.

## Geochemical evidence for Indian-type MORB and influence of surrounding subduction plates

IODP Expedition 349 [3] recovered, for the first time, basement oceanic crust samples near the fossil-spreading centers of the East Subbasin (ESB, Site U1431) and Southwest Subbasin (SWSB, Sites U1433 and U1434) (Fig. 1a). ^40^Ar/^39^Ar dating of these basement basalt samples revealed that the seafloor spreading of the ESB and SWSB terminated at a relatively close time range of ~15 and ~16–17 Ma, respectively [24], which is consistent with the interpretation of magnetic anomalies [25,26]. Most of the ESB basement samples are normal mid-ocean ridge basalts (N-MORBs) with a few showing enriched MORB (E-MORB) characteristics, while the SWSB samples are E-MORB [7]. MORB of both sub-basins shows an Indian Ocean-type isotopic mantle source (Fig. 1b) [7]. However, the SWSB samples are contaminated by 2–3% of lower continental crust as suggested by both isotopic data [7] and Fe_8.0_ and Na_8.0_ anomalies [8]. It was also suggested that the mantle potential temperature at the fossil ridge of the SCS might be higher than that beneath a normal mid-ocean ridge [27].

Increasing geochemical evidence points to the importance of subduction-induced mantle upwelling beneath the SCS. Volcanic rocks from the SCS seamounts are mostly oceanic island basalts of intermediate to mafic compositions [21,23]. Tholeiitic and alkalic basalts of the SCS and surrounding regions reveal a wide range of compositions, especially from recycled oceanic components [7,23]. The Hainan Island basalts show similar compositions to those of seamounts in the SCS and Indochina Peninsula [23]. The recovery of carbonate basalts [6] further strengthened the hypothesis of a recycled source for post-spreading magmatism.

## Geophysical evidence for influence of subducting plates

Geophysical evidence also points to interaction of the SCS-mantle dynamics with the surrounding subduction systems. Teleseismic tomography studies show that the Indian Plate has subducted at the Sumatra and Java trenches toward and beneath the SCS (Fig. 1a) to a depth of 800–1200 km [28,29]; meanwhile, seismicity studies of the Slab 2.0 model track the Indian slab to depths of at least 570 and 680 km, respectively, at the Sumatra and Java trenches [30]. The Philippine Sea Plate is subducting westward under the SCS to a depth of 600 km from the tomography model [28,29], while the seismicity-defined slab extends to a depth of at least 230 km [30]. Meanwhile, the Eurasian Plate is subducting eastward under the Philippine Sea Plate to a depth of ~400 km according to tomography studies [31,32], while the seismicity-defined slab extends to a depth of at least 170 km [30]. The above discussed broad zone of low seismic velocity under the northern SCS (Fig. 2a), together with the relatively high regional heat flow [33], is hypothesized to reflect a broad zone of mantle upwelling possibly induced by surrounding subducting plates. The melting associated with mantle upwelling likely caused the widespread post-spreading volcanism (Fig. 2b).

**Figure 3:**
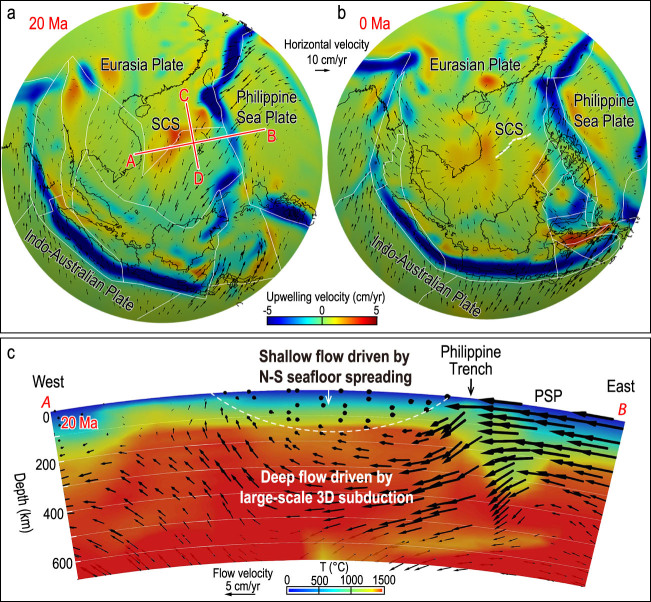
Calculated mantle upwelling velocity at a depth of 300 km at 20 Ma (a) and present (b). Also shown are velocity vectors at the corresponding depth relative to a moving hotspot reference frame [36]. (c) The E–W trending Profile A-B is parallel to the SCS-spreading axis at 20 Ma; profile location is shown in (a). Arrows show the calculated mantle-flow vectors and colors indicate the calculated mantle temperature. White dashed lines outline a shallow zone, in which the mantle flow is dominated by the N–S seafloor spreading of the SCS. The calculated velocity vectors within this shallow zone are found to point mostly out of the profile plane, as indicated by small black dots.

Whether the observed Hainan volcanism is caused by a mantle plume in a classic sense is a subject of continued debate [23]. Previous studies have envisioned that mantle plumes might originate from the core–mantle boundary [34,35] and are associated with massive eruptions within 2–3 Myr, ring-shaped picrite basalts of ultramafic compositions and dome-shaped crustal uplift. However, investigations of the Hainan region have not revealed the above features. The observed broad region of seismic anomalies above the MTZ (Fig. 2a) can be explained by broad mantle upwelling beneath the northern SCS rather than a narrow mantle plume.

## Geodynamic models linking plate subduction and mantle upwelling

Recent progress in geodynamic modeling is providing important new insights into the nature of mantle upwelling beneath the SCS and the relationship to surrounding subduction systems [9,10]. Here, we illustrate that some key observations discussed above could be explained by relatively simple geodynamic models in a self-consistent way (Fig. 3a).

**Figure 4:**
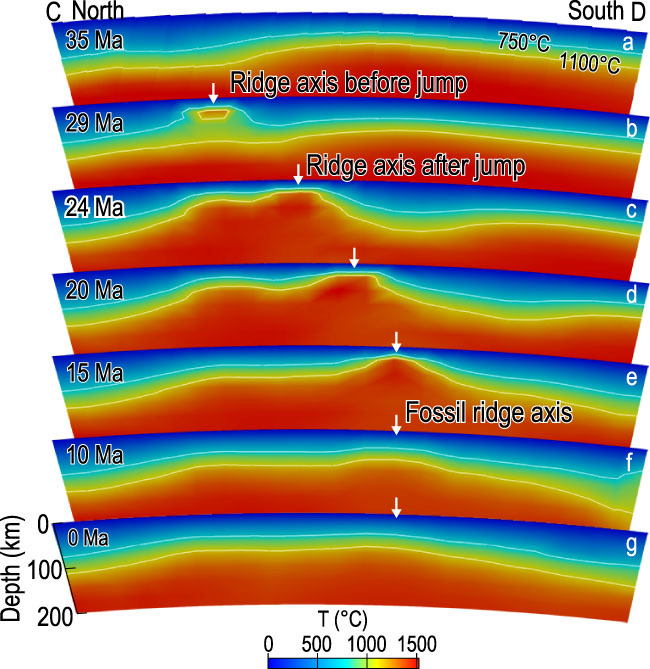
Calculated changes in mantle temperature along the N–S trending Profile C-D of the SCS, showing temperature changes with time. Profile location is shown in Fig. 3a.

We simulate the 3D flows of Navier-Stokes viscous fluid in a 2900-km-thick mantle layer in a spherical Earth with assumed constant temperatures at Earth’s surface (*T*_0_ = 0°C) and at the core–mantle boundary (*T*_m_ = 3500°C). The time-dependent mantle convection is driven by the observed kinematic motion of the surface tectonic plates since 160 Ma [36] and buoyant flows associated with lateral variations in mantle density. The mantle convection is simulated using the ASPECT (Advanced Solver for Problems in Earth’s ConvecTion) modeling platform [37]. The mantle viscosity is temperature- and depth-dependent; the reference value of a top 100-km-thick lid, as well as the lower mantle below 670 km, is 100 times that of the upper mantle (η_0_top_lid_ = η_0_lower_mantle_ = 100 η_0_upper_mantle_) [38]. Phase changes at the lower boundary of the MTZ were also considered [38]. The horizontal and vertical grid sizes are about 50 and 30 km, respectively, yielding a total of 50 million grid nodes for simulation of global-scale plate-driven mantle flows.

Modeling results reveal a layered structure of 3D mantle flows and two modes of mantle upwelling beneath the SCS: (i) linear but relatively narrow centers of mantle upwelling at shallow depths induced by divergent plate motion at seafloor-spreading centers and (ii) dome-shaped and relatively broad zones of mantle upwelling as a result of subduction-induced mantle-return flows at greater depths. These features are illustrated in an example at 20 Ma, when the SCS was undergoing N–S spreading (Fig. 3a). The calculated mantle-flow pattern and temperature structure are shown along the E–W trending Profile A-B, which is parallel to the SCS-spreading axis at 20 Ma (Fig. 3c).

*Mode 1*: Along this E–W trending profile (Fig. 3c), it is observed that the mantle flow at shallow depths is driven predominantly by the divergent plate motion associated with the N–S seafloor spreading (i.e. the shallow zone above the white dashed lines); such plate-driven shear flow is mostly limited to the top 100–200 km beneath the surface (Fig. 3c and Supplementary Fig. 3). During periods of SCS seafloor spreading at ~33–15 Ma, the *Mode 1* local mantle upwelling and high mantle temperature are calculated to exist beneath the spreading axis (Fig. 4b–d, Supplementary Fig. 1b–d and Supplementary Movie 1). After the cessation of the SCS seafloor spreading at ~15–17 Ma, the mantle temperature beneath the fossil ridge axis is calculated to decrease gradually (Fig. 4e–g and Supplementary Fig. 1e–h).

*Mode 2*: At greater depths, however, the mantle convection appears to be controlled predominantly by the E–W flow induced by the westward subduction of the Philippine Sea Plate at 20 Ma (Fig. 3c and Supplementary Fig. 3). Furthermore, a zone of relatively broad mantle upwelling is calculated to exist just to the west of the SCS-spreading axis at 20 Ma (Fig. 3a and c and Supplementary Fig. 3); such a broad zone of upwelling is calculated to have evolved and persisted until the present (Fig. 3b).

The synthesis of the above new observational constraints and geodynamic modeling points to an emerging new model of cycling of subduction-induced mantle flows in space and time. The new models of subduction-induced mantle-return flows provide a self-consistent framework for explaining some key observations of the SCS and for understanding the role of subduction zones in the formation and evolution of marginal seas [39]. At present, direct evidence for a narrow Hainan plume arising from the core–mantle boundary is still lacking. Instead, the observed broad region of seismic anomalies in the northern SCS could be explained by subduction-induced mantle flows. Such a broad zone of mantle upwelling could in turn help to explain the observed widespread post-spreading magmatism in the northern SCS and Indochina Peninsula. Future geodynamic modeling using quantitative geophysical and geochemical constraints is envisioned to provide further new insights into the SCS-mantle geodynamics.

## Supplementary Material

Supplementary_figures-Lin_et_al-NSR-2019-182_nwz123

Supplementary_movie-Lin_et_al-NSR-2019-182_nwz123
