# Supplementary material for: Mantle upwelling beneath the South China Sea and links to surrounding subduction systems
Source: Natl Sci Rev. 2019 Aug 28;6(5):877–81. doi: 10.1093/nsr/nwz123 (PMC8291476; doi:10.1093/nsr/nwz123)
Supplement: Supplementary_figures-Lin_et_al-NSR-2019-182_nwz123 [file supplementary_figures-lin_et_al-nsr-2019-182_nwz123.docx]

**Supplementary Information**

Special Topics: New Discovery of the South China Sea Ocean Drilling

**Mantle upwelling beneath the South China Sea and links to surrounding subduction systems**

Jian Lin^1, 2, *^, Yigang Xu^3^, Zhen Sun^2^, Zhiyuan Zhou^2^

^1^ Department of Geology and Geophysics, Woods Hole Oceanographic Institution, USA

^2^ Key Laboratory of Ocean and Marginal Sea Geology, South China Sea Institute of Oceanology, Chinese Academy of Sciences, China

^3^ State Key Laboratory of Isotope Geochemistry, Guangzhou Institute of Geochemistry, Chinese Academy of Sciences, China

***Corresponding author**.

Email: jlin@whoi.edu

Note: This document contains the following supplementary data:

1. Supplementary Figure 1.
2. Supplementary Figure 2.
3. Supplementary Figure 3.
4. Supplementary Movie1.


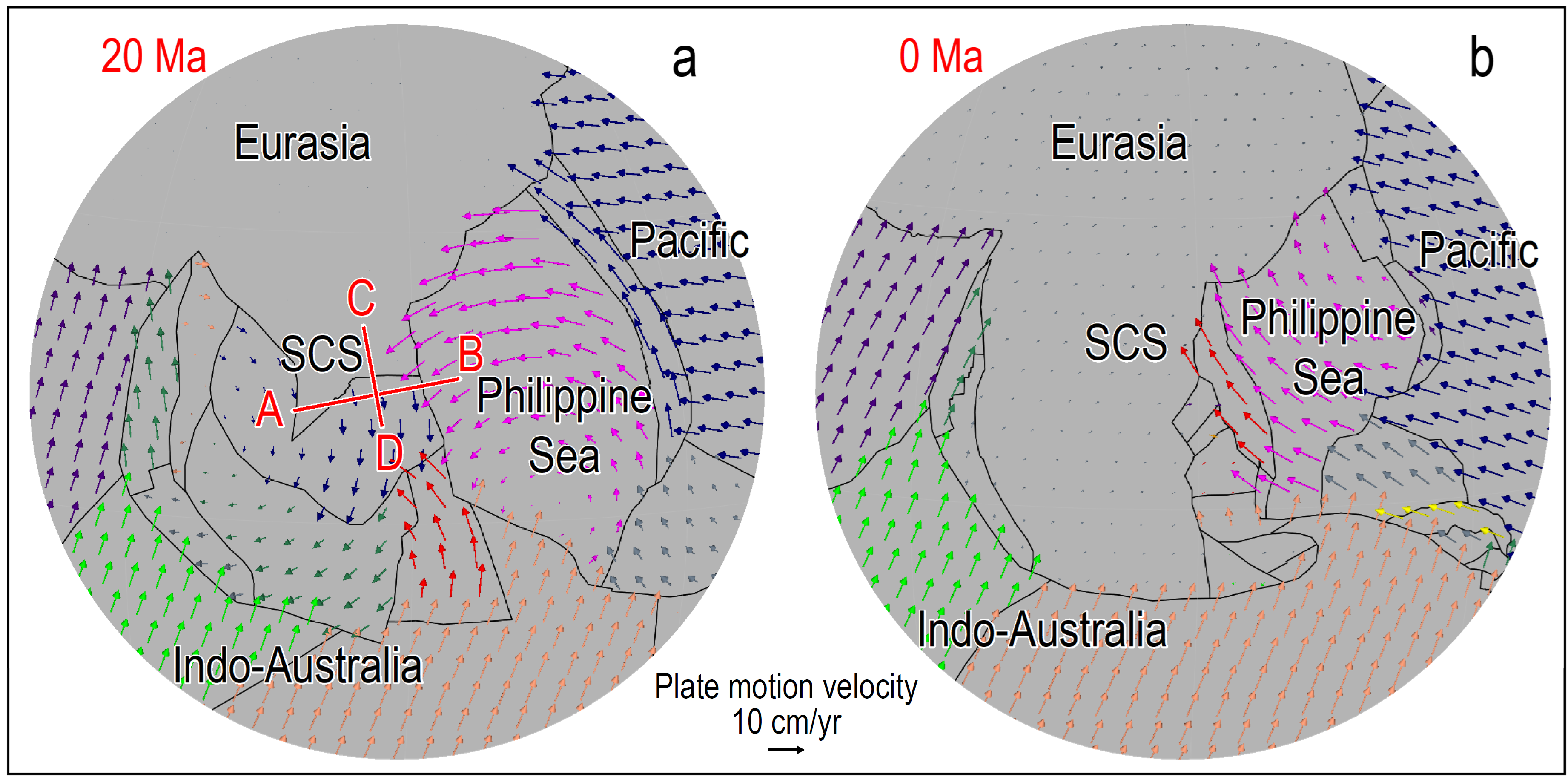


**Supplementary Figure 1.** Velocity vectors of the surface plates relative to a moving hotspot reference frame at 20 Ma (a) and present (b). The velocity solutions were obtained from GPlates and used as suface boundary conditions in ASPECT modeling of the 3D mantle flow.


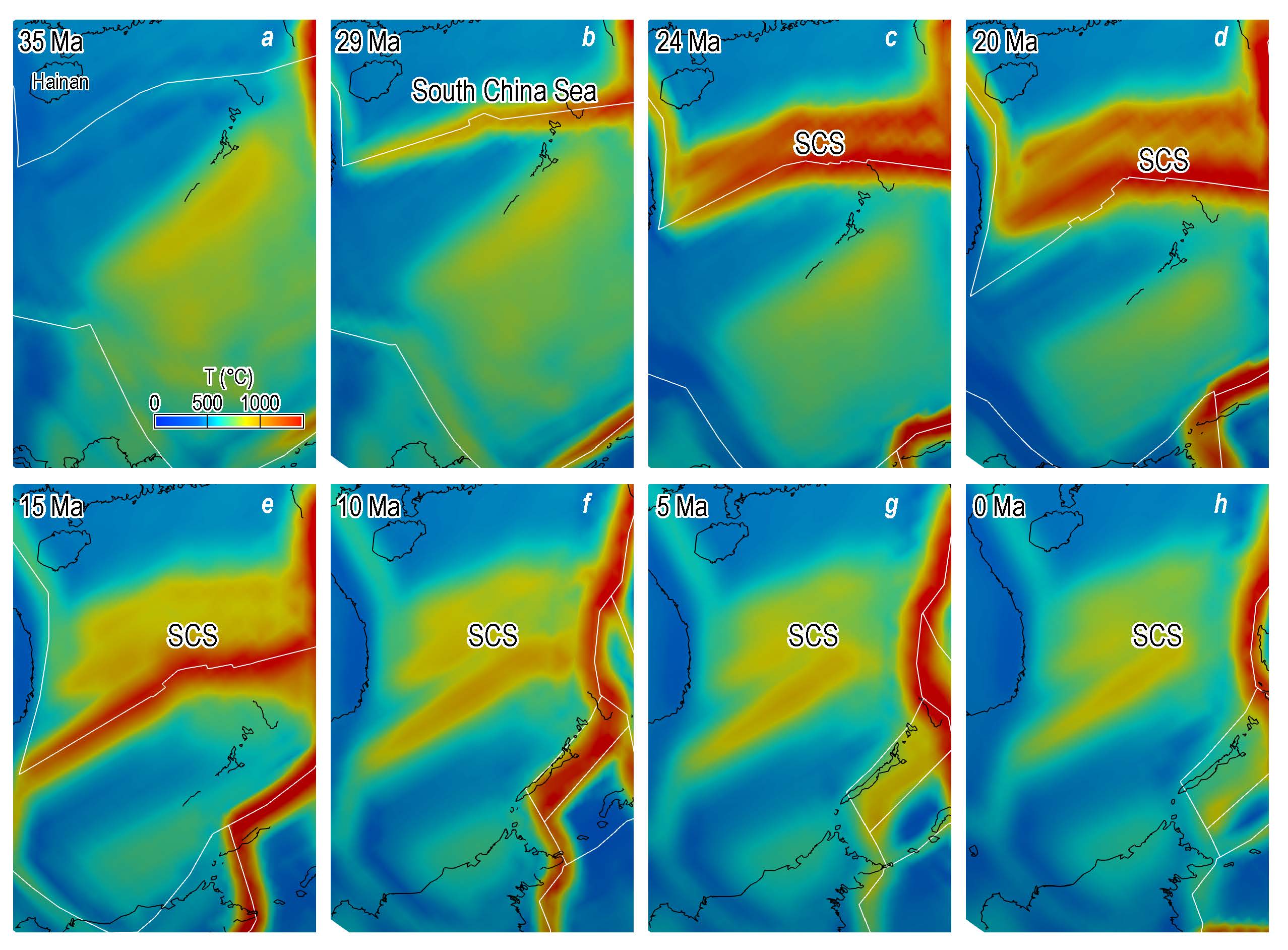


**Supplementary Figure 2**. Calculated mantle temperature at a depth of 50 km from 35 Ma to present.


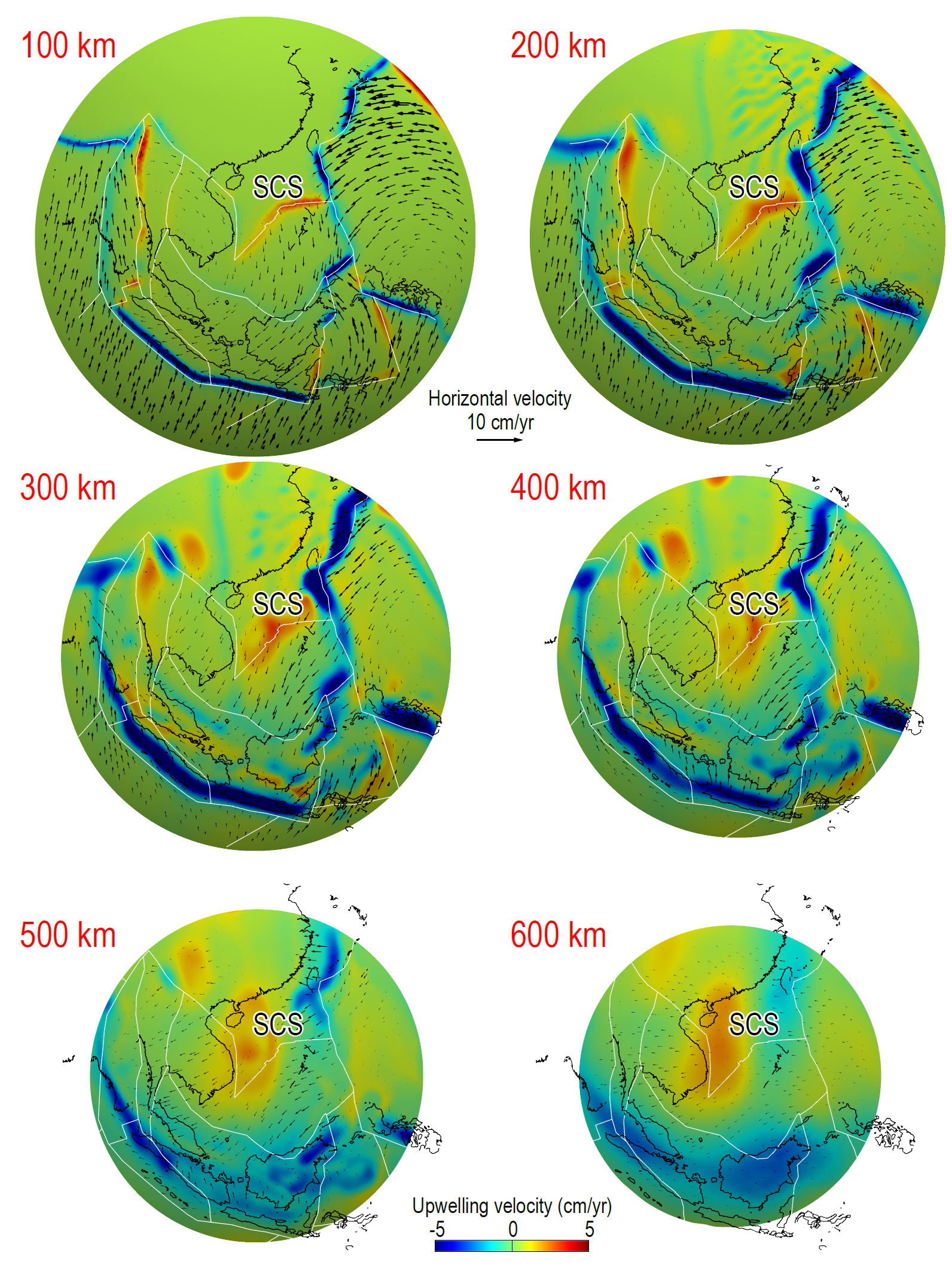


**Supplementary Figure 3**. Calculated mantle upwelling velocity at the depths of 100 to 600 km beneath the study area at 20 Ma. Black arrows show calculated horizontal veoclities at corresponding depths relative to a moving hotspot reference frame. Black and white curves indicate coastlines and plate boundaries, respectively.

**Supplementary Movie1**. Calculated mantle temperature at depth of 50 km from 100 Ma to present.
